# Graft tunnel integration occurs early in the tibial tunnel compared with the femoral tunnel after anterior cruciate ligament reconstruction with preserved insertion hamstring tendon graft

**DOI:** 10.1186/s43019-021-00119-x

**Published:** 2021-10-09

**Authors:** Ravi Gupta, Sandeep Singh, Anil Kapoor, Ashwani soni, Ravinder Kaur, Narinder Kaur

**Affiliations:** 1 Unit II, Orthopaedics Cum Project Director Sports Injury Centre and Medical Superintendent, Government Medical College Hospital, Chandigarh, India; 2 Department of Orthopaedics, Government Medical College Hospital, Chandigarh, India; 3 Department of Radio-Diagnosis, Government Medical College Hospital, Chandigarh, India

**Keywords:** Hamstring tendon graft with preserved insertion, Graft integration, Tunnel integration, ACL

## Abstract

**Background:**

Preservation of hamstring tendon insertion at the time of anterior cruciate ligament (ACL) reconstruction is a well-known technique; however, its effect on graft integration is not well studied. The present study was conducted to study the graft integration inside the tibial and femoral tunnels, respectively, after ACL reconstruction using hamstring tendon graft with preserved insertion.

**Methods:**

Twenty-five professional athletes who underwent ACL reconstruction using hamstring tendon graft with preserved tibia insertion were enrolled in the study. Functional outcomes were checked at final follow-up using Lysholm score and Tegner activity scale. Magnetic resonance imaging (MRI) was done at 8 months and 14 months follow-up to study the graft tunnel integration of the ACL graft at both tibial and femoral tunnels.

**Results:**

The mean Fibrous interzone (FI) score (tibial tunnel) decreased from 2.61 (1–5) at 8 months to 2.04 (1–4) at 14 months follow-up (*p* = 0.02). The mean FI score (femoral side) decreased from 3.04 (2–5) at 8 months to 2.57 (2–4) at 14 months (*p* = 0.02).

**Conclusions:**

Graft integration occurs early in the tibial tunnel as compared with the femur tunnel with preserved insertion hamstring tendon autograft.

*Trial registration* CTRI/2019/07/020320 [registered on 22/07/2019]; http://www.ctri.nic.in/Clinicaltrials/pdf_generate.php?trialid=33884&EncHid=&modid=&compid=%27,%2733884det%27

## Introduction

Anterior cruciate ligament (ACL) reconstruction is a commonly performed orthopedic procedure [1]. The goal of ACL reconstruction is to restore knee stability, reduce the risk of secondary meniscal and chondral lesions, and allow a safe return to sports and athletic activities [2, 3]. Hamstring tendon graft and bone–patellar tendon–bone graft are the two most commonly used autografts for ACL reconstruction [4]. Hamstring tendon grafts can be used as a free graft (semitendinosus and gracilis, STG) or preserved insertion graft (STGPI).

The hamstring tendon graft is a soft tissue graft that needs to be integrated within the bone tunnels (graft tunnel integration) [5]. Graft integration is important for the restoration of the native knee kinematics [6]. Studies have shown that, after ACL reconstruction, a cell- and vessel-rich fibrous interzone (FI) forms between the tendon graft and the bone tunnel wall [7, 8]. This interface consists of disorganized, highly cellular, and highly vascular connective granulation tissue during the early healing phase. With further healing, the FI tissue gets less cellular and vascular and there is an early development of Sharpey-like collagen fibers bridging the interface, and the amount of collagen fibers increases over time [9]. Graft tunnel healing is a complex process that can be affected by several factors such as the type of graft, length of the graft, preserved muscle fibers, preserved vascularity of the graft, hyperbaric oxygen treatment to graft, thermal damage caused by drill, etc. [10–14].

Graft tunnel integration is an important factor affecting graft maturation, and hence it is crucial for the proper functioning of reconstructed ACL. Therefore, the present study was conducted to compare the graft integration inside the femoral and tibial tunnel after ACL reconstruction using STGPI graft.

It was hypothesized that preservation of hamstring tendon insertion hastens the process of graft integration in the tibial tunnel.

## Methods

This was a prospective study conducted in a regional sports injury center after institutional ethical committee approval (GMC/12C/2018/277). The present study is registered with the Clinical Trials Registry-India (CTRI/2019/07/020320). Twenty-five elite male sportspersons of age 18–35 years who underwent ACL reconstruction using STGPI graft were enrolled in the study. Exclusion criteria were female patients, multi-ligament injury, history of chronic inflammatory disease, previously operated on the same knee, smokers, alcohol, and history of steroid intake.

Data for this study were collected prospectively from 25 patients involved in different sporting activities who had undergone primary ACL reconstruction. ACL reconstruction was done using a quadruple hamstring tendon graft using the transportal technique. Both the semitendinosus and gracilis tendon insertions at the tibia were preserved, and their free ends were sutured back to the insertion site [15]. Endobutton was used at the femoral end for fixation of the graft. Fourteen of the 25 patients had meniscal tear, for which meniscal repair was done in 2 patients, and the remaining 12 patients were treated by partial meniscectomy.

### Surgical technique: ACL reconstruction using STGPI graft

The semitendinosus and gracilis (STG) grafts were harvested through a 3–5 cm incision centered 2 cm medial to the tibial tuberosity. The proximal free ends of the tendons were sutured together using Ethibond no. 5 suture (Ethicon Inc., Johnson and Johnson, India, Mumbai). The tendons were looped around an Ethibond No. 5 suture placed at their middle, thus creating a quadrupled graft. The graft was sized with graft sizers of 0.5 mm increments.

**Fig. 1:**
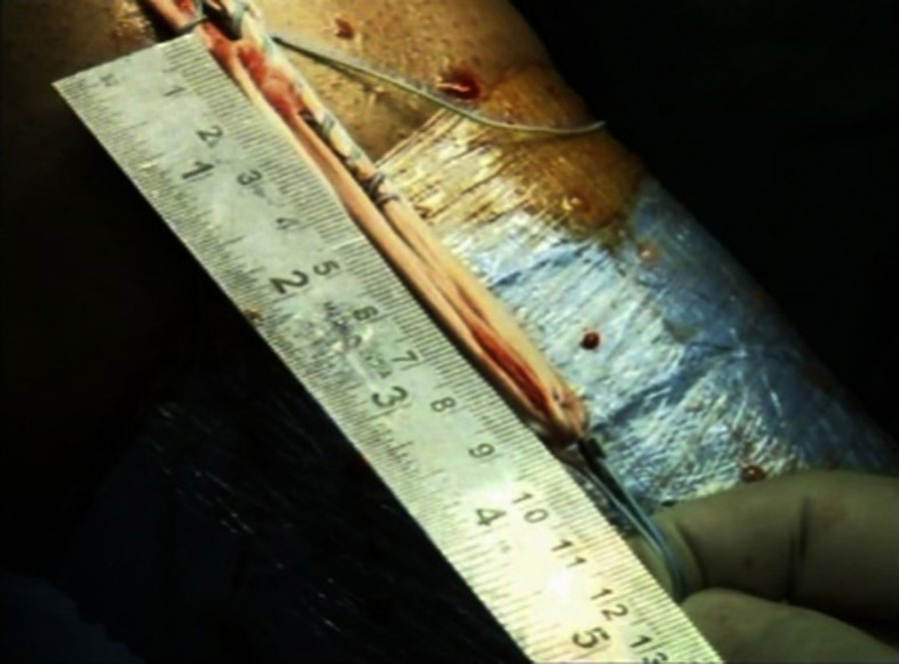
The length of quadruple graft plus endobutton loop was kept equal to length of tunnels plus intraarticular part

**Fig. 2:**
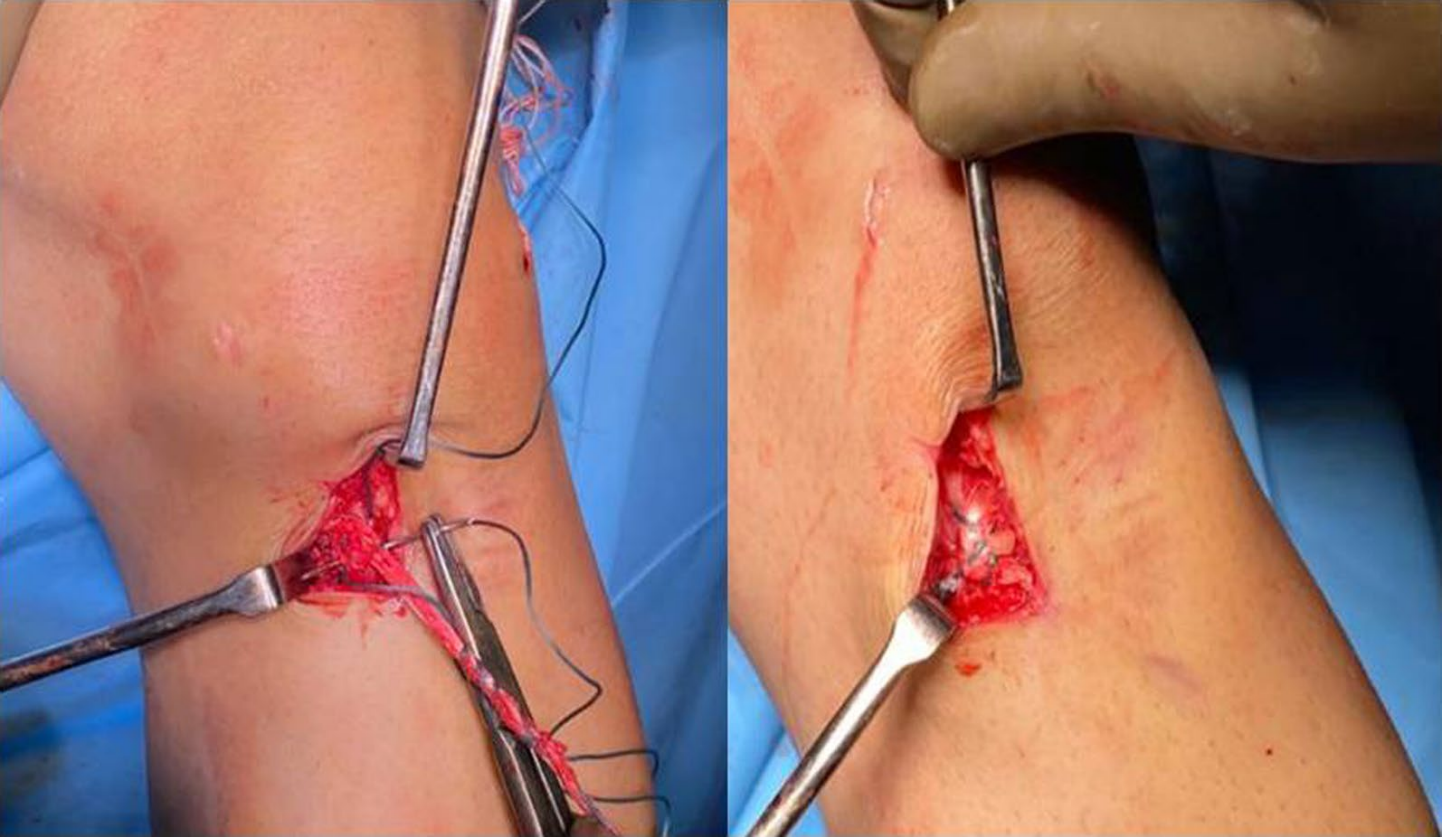
The free end of the quadruple graft was sutured back to insertion of hamstring tendon with Ethibond no. 5

The femoral tunnel was drilled using the transportal technique. A 4.5 mm canulated drill bit was used to create a tunnel in the femur. The length of the tunnel was measured with depth gauze. The reaming of the tunnel was done with a femoral reamer corresponding to the diameter of the graft. The no. 5 Ethibond was passed into the tunnel, and the loop was parked inside the joint. The tibial tunnel was drilled using a tibial tunnel guide (Smith and Nephew India Ltd.) with the angle of the guide kept at 55°. A tibial reamer with a diameter equivalent to the size of the graft was used to drill the tunnel. The loop of the Ethibond no. 5 parked inside the knee joint was retrieved from the tibial tunnel with the help of a suture grasper. The length of the tibial tunnel and the intraarticular part of the proposed graft was measured with a depth gauge, which was added to the already measured length of the femoral tunnel to determine the exact length of both the tunnels plus the intraarticular part of the graft. An endobutton was selected so that at least 15 mm of the graft remained inside the femoral tunnel. Quadruple graft length plus loop length was kept equal to total tunnel length plus intraarticular length (Fig. 1). The free end of the graft was pulled to maximal stretch. With maximal stretch on the free end of the graft, it was sutured to the preserved end of the graft with a no. 5 Ethibond (Fig. 2).

### Rehabilitation protocol

On postoperative day 0, knee range of motion and static quadriceps exercises were started as per pain tolerance. From postoperative day 1, straight leg raising and full weight-bearing with brace were started. This regimen was continued for up to 6 weeks. At 6 weeks, the use of brace was discontinued and static cycling and half squatting were introduced in addition to the existing physiotherapy. At 3 months, light jogging and full squatting were allowed. During 3–6 months, patients underwent conditioning, endurance, proprioception enhancement, and sport-specific training exercises. After 6 months of the rehabilitation program, limb symmetrical index (operated knee/normal contralateral knee) was calculated using single hop test, thigh wasting, and knee laxity. If the limb symmetrical index (LSI) was more than 85%, athletes were allowed to return to sports in practice games and subsequently to professional matches; however, if LSI was less than 85% in any of the parameters, players were asked to continue physiotherapy and revisit monthly till LSI > 85% was achieved.

Knee laxity was checked by anterior drawer test and Lachman test at 8 months and 14 months.

MRI was done at 8 months and 14 months follow-up to study the graft tunnel integration of ACL graft in both the tibial and femoral tunnels. The MRI follow-up time was decided after considering the timing of return to sports. All the patients in the present study were professional athletes, and previous studies have shown that the mean time to return to sports was around 8–9 months [16, 17]. Therefore, the first MRI was done at 8 months. Secondly, the usual time of graft maturation is around 1–2 years, therefore, the second MRI was done at 14 months, and by 12–15 months most patients achieved final functional outcomes and further improvement is unlikely beyond this period. The functional status of all the patients was recorded using the Lysholm knee scoring scale, Tegner activity scale, and return to sports.

### MRI methodology

In the present study, MRI was performed on Achieva 1.5 T model (Phillips Medical Systems) using a standard polarized knee coil supplied by the manufacturer. Coronal and sagittal short tau inversion recovery (STIR) imaging with 3 mm slice thickness with 0.3 mm gap with field of view (FOV) 252 × 154 mm and number of signal averages (NSA) 1.9 were obtained. The sagittal images were placed along the longitudinal axis of the ACL graft using an axial scout view. Three-millimeter slice thickness with 0.3 mm gap, one acquisition, and a 504 × 237 matrix was used. Total imaging time for this protocol was 12–20 min. The local ethics committee approved the MRI study protocol.

**Fig. 3:**
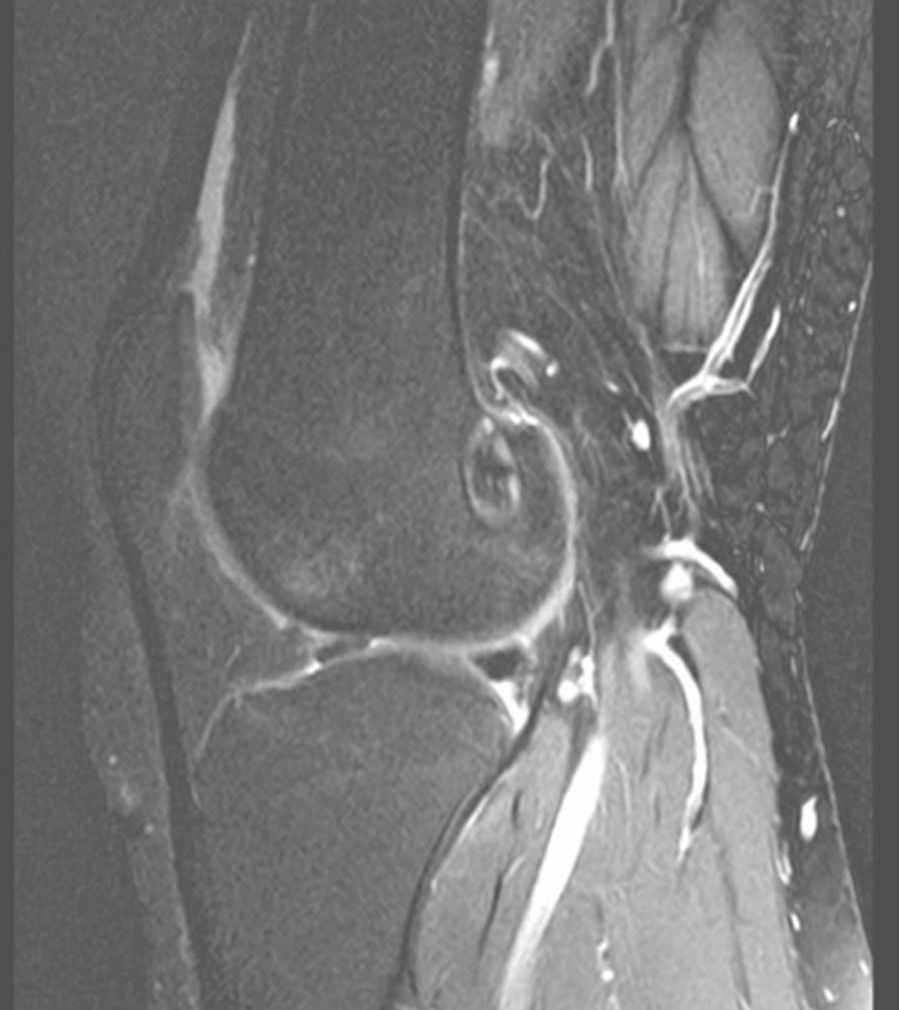
Sagittal magnetic resonance imaging of the fibrous interzone in the femoral tunnel in STGPI graft

**Fig. 4:**
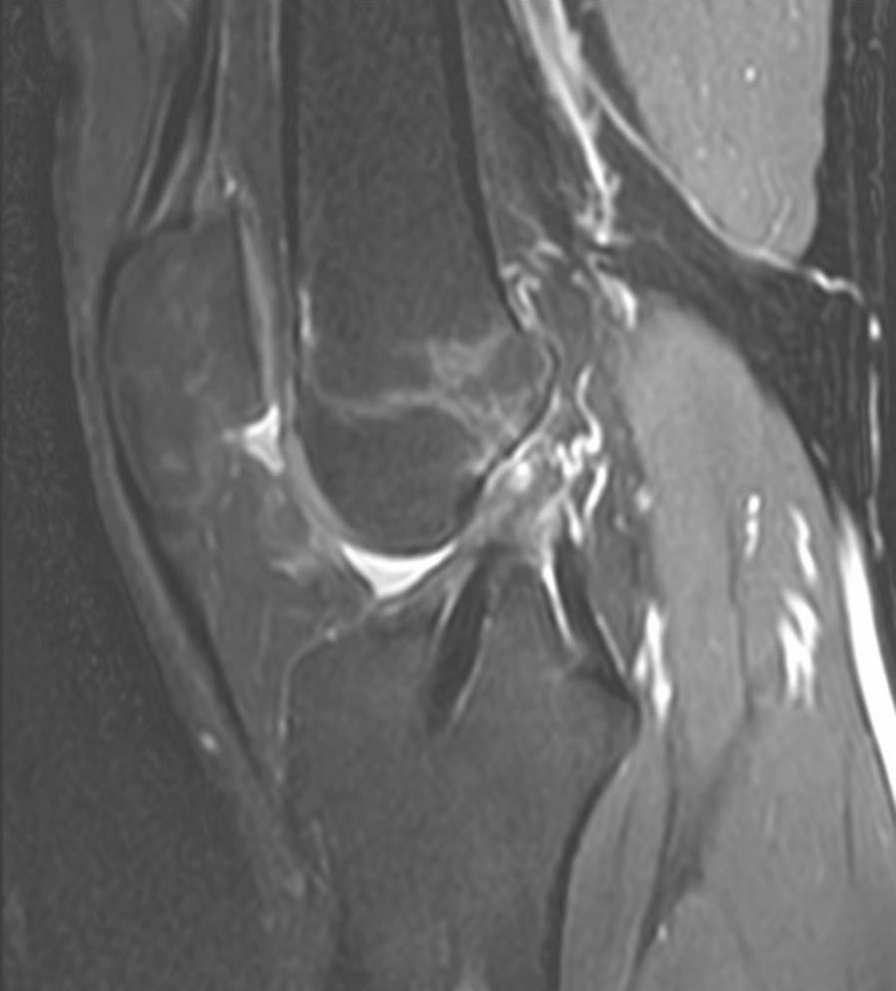
Sagittal magnetic resonance imaging of the fibrous interzone in the tibial tunnel in STGPI graft

**Fig. 5:**
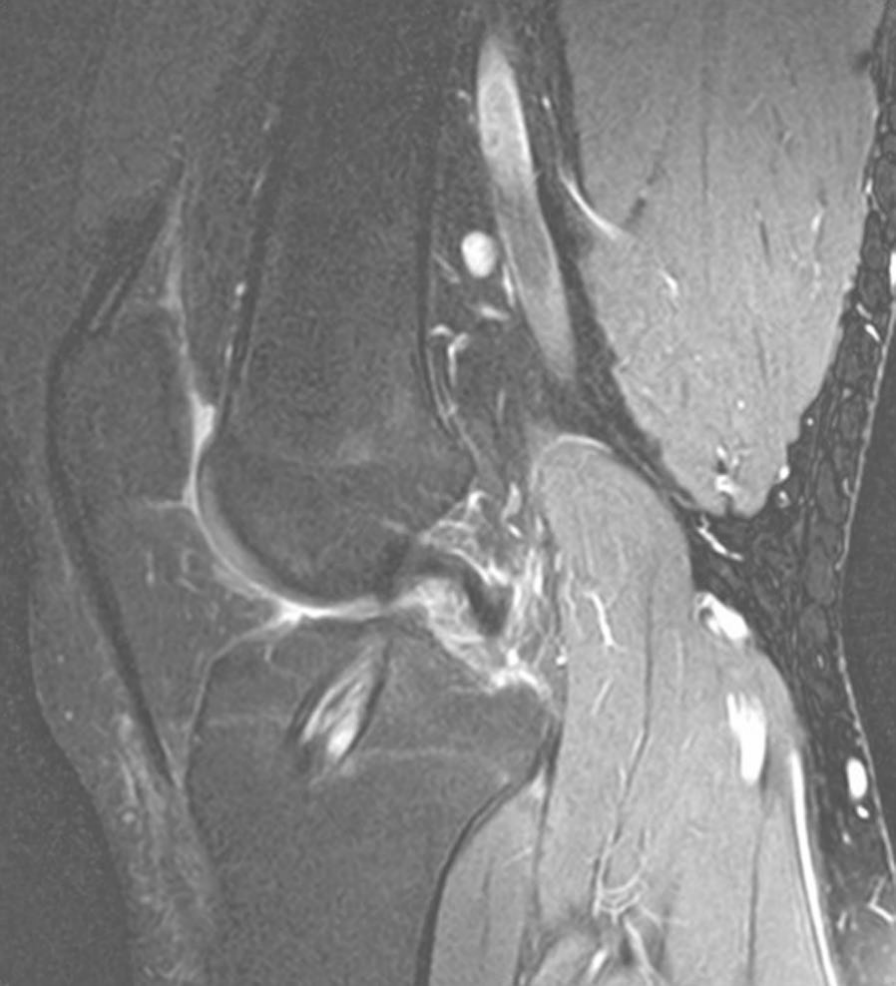
Grade 1 stage (FI score) of graft integration

The images were processed and evaluated on OsiriX software for the Apple Mac system. The MR images were interpreted by a consensus of two readings, and the following findings were recorded:

Graft tunnel integration was analyzed by visually assessing the signal intensity of the fibrous interzone on sagittal STIR image (Figs. 3, 4, and 5), and scoring was done based on its comparison with the anatomical landmarks. The signal intensity was given a score of 1 (similar to the patellar tendon), 2 (greater than patellar tendon but less than muscle), 3 (similar to muscle), 4 (greater than muscle but less than joint fluid), and 5 (similar to joint fluid) [18].

### Statistical analysis

Data were coded and recorded in the MS Excel spreadsheet program. SPSS v23 (IBM Corp.) software was used for data analysis. Descriptive statistics were elaborated in the form of means/standard deviations and medians/interquartile ranges (IQRs) for continuous variables, and frequencies and percentages for categorical variables. Correlation of FI score with timing and site (tibial and femoral) was assessed using the Wilcoxon test. Statistical significance was determined at *p* < 0.05.

## Results

The mean age of the patients in the present study was 25.1 ± 5.1 years. The mean BMI was 23.8 kg/m^2^. Eleven out of 25 patients had dominant limb involvement, and 14/25 patients had nondominant involvement.

Nonparametric tests were used to make a statistical inference as data were not normally distributed. Paired Wilcoxon test was used to explore the difference in fibrous interzone score at the two time points. The mean FI score (tibial tunnel) decreased from 2.61 (1–5) at 8 months to 2.04 (1–4) at 14 months follow-up (Wilcoxon test: *V* = 69.0, *p* = 0.017). The mean FI score (femoral side) decreased from 3.04 (2–5) at 8 months to 2.57 (2–4) at 14 months (Wilcoxon test: *V* = 67.0, *p* = 0.021).

On comparing the graft tunnel integration in femoral and tibia tunnel (Table 1), it was observed that, at 8 months follow-up, the mean FI score at the tibial end was 2.61 ± 1.34 and at the femoral end was 3.04 ± 1.02 (Wilcoxon test: *V* = 0.0, *p* = 0.010). At 14 months follow-up, mean FI score at tibial end was 2.04 ± 0.82 and at femoral end was 2.57 ± 0.66 (Wilcoxon test: *V* = 0.0, *p* = 0.004).

**Table 1:** The mean FI scores at femoral and tibial tunnel at 8 months and 14 months

Mean FI score	At 8 months	At 14 months	*p*-Value
Tibial tunnel	2.6 (1–5)	2.08 (1–4)	0.02
Femoral tunnel	3.04 (2–5)	2.57 (2–4)	0.02
*p*-Value	0.01	0.004	

The mean graft diameter was 7.8 ± 06 mm. The mean FI score (tibia) at 14 months was 1.55 in patients having graft diameter ≤ 7.5 (*n* = 11) and 2.4 in patients having graft diameter > 7.5 (*n* = 14; *p* = 0.008). The mean femur FI score at 8 months was 2.45 in patients having graft diameter ≤ 7.5 (*n* = 11) and 2.64 in patients having graft diameter > 7.5 (*p* = 0.5).

The mean Lysholm score (Table 2) in patients with FI score (tibia) ≤ 2 and FI score ≥ 3 was 94.2 and 89.2, respectively (*p* = 0.03). Similarly, the mean difference in pre-injury and post-surgery (14 months) Tegner activity scale in patients with FI score (tibia) ≤ 2 and FI score ≥ 3 was 0.3 and 1.3, respectively (*p* = 0.02). The mean Lysholm score (Table 3) in patients with FI score (femur) ≤ 2 and FI score ≥ 3 was 94.4 and 90.2, respectively (*p* = 0.047).

**Table 2:** Comparison of functional outcomes according to FI score at tibia

FI score at 14 months at tibia	Total number of patients	Mean Lysholm score	Mean pre-injury Tegner activity scale	Mean Tegner activity scale	Percentage of patients return to sports
≥ 3	8	89.2	7.7	6.4	3 (37.5%)
≤ 2	17	94.2	7.7	7.2	13 (76%)
*p*-Value		0.03	1	0.02	0.08

**Table 3:** Comparison of functional outcomes according to FI score at femur

FI score at 14 months at femur	Total number of patients	Mean Lysholm score	Mean pre-injury Tegner activity scale	Mean Tegner activity scale	Percentage of patients return to sports
≥ 3	11	90.2	7.7	6.7	5 (45%)
≤ 2	14	94.4	7.6	7.2	11 (79%)
*p*-Value		0.047	0.8	0.2	0.1

## Discussion

The main finding of the present study was that graft incorporation happened early in the tibial tunnel compared with the femoral tunnel. Many factors can affect the graft tunnel integration such as type of graft [autograft versus allograft; bone–patellar tendon–bone (BPTB) versus hamstring graft], size of the graft, fixation method, preservation of muscle fiber, BMPs, etc. [19, 20]. Hamstring tendon grafts have gained more popularity due to less donor site morbidity as compared with BPTB grafts. However, hamstring tendon graft requires the critical biological process of bone-to-tendon integration to be undergone as compared with the bone-to-bone healing in BPTB graft. The estimated time for graft tunnel integration is 6 weeks for the BPTB graft and 8–12 weeks for the hamstring tendon graft [21, 22]. This is a very critical step as sufficient bone–tendon integration is important for return to sports activities [23]. Hon-Yun et al. also observed that tendon-to-bone healing has a direct correlation with functional outcomes [24]. However, Martin et al. reported that marginal articular surface graft healing is more important than intratunnel healing [25].

Ruffilli et al. in a meta-analysis study concluded that, despite promising results of preserving the hamstring tendon insertion during anterior cruciate ligament reconstruction (ACLR), a prospective MRI evaluation study was required [26]. Therefore, the present study was conducted to document the effect of the preservation of hamstring tendon graft on graft tunnel integration. However, the present study was a case series study and results were not compared between the free graft and preserved insertion graft; therefore, there is a need for a large cohort comparative study, and the present study can act as a base for such studies in the future.

The possible reason for early graft tunnel integration in the tibial tunnel compared with the femoral tunnel could be preserved hamstring tendon tibial insertion and the presence of remnant muscle fibers. In preserved insertion ACLR, blood supply at insertion end is preserved, which could facilitate early graft incorporation. Secondly, despite the best efforts of removing muscle fibers from the tendon, there are always some fibers that remain attached to the tendon, and the presence of these muscle fibers facilitates graft tunnel integration. Cuti et al. [27] examined the capacity of muscle-derived stem cells harvested from hamstring tendon and found that muscle cells expanded faster, exhibited more alkaline phosphatase activity, and had higher expression of bone sialoprotein than tendon cells. Landry et al. [28] studied the periosteal response to skeletal trauma when the muscle was also injured and found that muscle injury increased proliferation in the periosteum and induction of osteoblasts during the early injury stages. Junsuke et al. also observed that grafted tendon healing occurs early in the tibial tunnel compared with femoral tunnel; they suggested that the differences in mechanical (stress) pressure inside the tunnel can be the cause of this differential healing [7].

In the present study, it was observed that patients with better graft integration had better clinical outcomes (Table 2; Lysholm score and Tegner activity scale). Hon-Yun et al. also observed that tendon-to-bone healing has a direct correlation with functional outcomes [24]. However, Martin et al. reported that marginal articular surface graft healing is more important than intratunnel healing [25]. The results of the present study suggest that graft tunnel integration has a positive correlation with functional outcomes.

The present study had some limitations. First, this study may have been underpowered (type 2 error) to detect the correlation between the graft integration and functional outcomes, as the study was powered to detect the graft integration inside the tunnels; therefore, further studies are required to establish the correlation between graft integration and functional outcomes. Second, there was no control group in the present study; therefore, comparative study between STG free graft and preserved insertion graft will be needed in the future. Third, all the patients in the present study were males; therefore, the effect of gender on graft integration could not be studied. Fourth, the effect of graft fixation methods on the graft tunnel integration was not studied; in the present study, the femoral end of the graft was fixed using endobutton, and the tibial end of the graft was sutured back to the insertion. Although previous studies have observed that this method of fixation result in satisfactory functional outcomes [29], 30], there are not many biomechanical studies that compared these different methods of fixation. Therefore, studies are required in the future to see the effect of graft fixation methods on graft integration.

## Conclusions

Graft tunnel integration occurs early in the tibial tunnel compared with the femoral tunnel after anterior cruciate ligament reconstruction with preserved insertion hamstring tendon graft. Clinical outcomes after ACL reconstruction are directly correlated to graft tunnel integration. Level of study—4.

## Data Availability

The datasets during and/or analyzed during the current study are available from the corresponding author on reasonable request.
